# Hole‐Accepting‐Ligand‐Modified CdSe QDs for Dramatic Enhancement of Photocatalytic and Photoelectrochemical Hydrogen Evolution by Solar Energy

**DOI:** 10.1002/advs.201500282

**Published:** 2015-12-02

**Authors:** Xu‐Bing Li, Bin Liu, Min Wen, Yu‐Ji Gao, Hao‐Lin Wu, Mao‐Yong Huang, Zhi‐Jun Li, Bin Chen, Chen‐Ho Tung, Li‐Zhu Wu

**Affiliations:** ^1^ Key Laboratory of Photochemical Conversion and Optoelectronic MaterialsTechnical Institute of Physics and ChemistryThe Chinese Academy of SciencesBeijing100190P. R. China

**Keywords:** artificial photosynthesis, charge transfer, H_2_ evolution, hole transfer, ligand‐modified QDs

## Abstract

**Solar H_2_ evolution of CdSe QDs** can be significantly enhanced simply by introducing a suitable hole‐accepting‐ligand for achieving efficient hole extraction and transfer at the nanoscale interfaces, which opens an effective pathway for dissociation of excitons to generate long‐lived charge separation, thus improving the solar‐to‐fuel conversion efficiency.

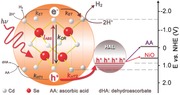

Production of molecular hydrogen (H_2_) from water is one of the most promising avenues to convert solar energy into clean and sustainable fuels.^1^ Over the past several years, this straightforward transformation has spurred tremendous research efforts, and there have been numerous advances in understanding the mechanism involved in the process of proton reduction, and in designing organometallic complexes as well as biomimics based on earth‐abundant elements for photochemical and electrochemical H_2_ evolution.^2^ Owing to unique characteristics amenable to applications in light harvesting and charge separation, quantum dots (QDs) have been recently demonstrated as promising candidates for solar H_2_ evolution.^3^ Their size‐dependent optical properties and large extinction coefficients over a broad spectral range dramatically improve solar light‐harvesting performance, and their quantum confinement effects and large surface‐to‐volume ratios significantly enhance the surface amplitude of photogenerated electrons and holes.^4^ For these reasons, QDs alone or coupled with external co‐catalysts have shown much higher efficiencies for solar H_2_ evolution than organometallic complexes or bulk semiconductor counterparts.^5^ Better understanding the key factors that govern the efficiencies of QD‐based artificial photosystems for solar H_2_ evolution would be of significance to take one step further toward water splitting to be commercially viable.

It has been well established that the overall process of solar H_2_ evolution involves three fundamentals.^6^ The first is light capture: absorbing sunlight by QDs. The second is exciton‐generation: pushing sunlight‐excited electrons off their home atoms to form spatially separated electron/hole pairs in the valence and conduction bands. And the third is catalysis: the generated holes are captured by sacrificial electron donors, and the electrons are utilized for proton reduction. Spectroscopic studies have revealed that photoinduced electron transfer from QDs to catalytic sites can occur smoothly, but hole extraction of QDs is somewhat problematic.^7^ As the rate of hole transfer is about two to four orders of magnitude slower than that of electrons,^8^ hole removal is regarded as a key efficiency‐determining step for photocatalytic and photoelectrochemical (PEC) H_2_ evolution in QD‐based artificial photosystems.^9^ Recently, there is a growing interest in modifying QDs with specific ligands to increase inter‐particle charge transport and exciton delocalization at the interface of inorganic core and organic‐ligand layer.^10^ In this regard, one may expect that introducing smart ligands (ligands with excellent hole‐accepting ability) to overcome the hole‐removal limitation of QDs would offer new opportunities to improve the efficiency of solar H_2_ evolution.

In this contribution, we wish to report that merely introduction of hole‐accepting ligands onto the surface can significantly enhance the photocatalytic H_2_ production of CdSe QDs in aqueous solution and PEC H_2_ evolution of CdSe QDs‐sensitized photocathode from neutral water. A suitable hole‐accepting ligand should satisfy the energy requirement for successive hole transfer and interact with CdSe QDs intimately to make ultrafast hole transfer feasible. Phenothiazine (PTZ) is likely to serve as an ideal ligand for such application because the highest occupied molecular orbital (HOMO) level of PTZ (≈0.9 V vs NHE) is more negative than the valence band of CdSe QDs (≈1.3 V vs NHE;^11^ ≈1.9 nm in diameter), implying that hole transfer from CdSe QDs to PTZ is exothermic. Moreover, the electron‐rich heterocyclic structure of PTZ not only allows ready electron‐donating ability but also ensures intimate interaction with QDs through sulphur‐cadmium coordination and van der Waals interaction.^12^ Indeed, the rate of visible‐light‐induced H_2_ evolution of PTZ‐modified CdSe QDs in a typical artificial photosystem containing ascorbic acid was increased to ≈7500 μmol h^−1^ g^−1^, ≈40‐fold to that of bare CdSe QDs. More importantly, PTZ modification of CdSe QDs could significantly enhance the transient photocurrent response, the maximal incident photon‐to‐current conversion efficiency (IPCE) and PEC H_2_ evolution efficiency of CdSe QDs‐sensitized photocathode from neutral water to ≈180 μA cm^−2^, ≈10.3% and ≈3000 μmol h^−1^ g^−1^ cm^−2^ respectively under a combination of visible‐light irradiation and applied bias of −0.1 V versus NHE in a three‐electrode system, ≈2.5 fold to those obtained in the absence of PTZ.

Synthesis of thiol stabilized CdSe QDs was described in our previous reports.^13^ The as‐synthesized CdSe QDs were characterized by steady‐state spectra (Figure S1a, Supporting Information) and high‐resolution transmission electron microscopy (TEM) (Figure S2, Supporting Information). PTZ‐modified CdSe QDs were prepared through ligand exchange, see details in the Experimental Section. Successful introduction of PTZ onto the surface of CdSe QDs could be verified by Fourier transform infrared spectroscopy (Figure S3, Supporting Information). Solar H_2_ evolution experiment was carried out by dispersing PTZ‐modified CdSe QDs (1.6 × 10^−5^ mol L^−1^) in a Pyrex tube containing 10.0 mL aqueous solution of ascorbic acid (0.2 m) at pH 4.0. The solution was deaerated by N_2_ for 30 min to remove the residual O_2_ and then irradiated under a 500 W high‐pressure mercury lamp (≈100 mW cm^−2^ near the surface of lamp) with a cut‐off filter to remove light below 400 nm. Amount of evolved H_2_ at the headspace of the Pyrex tube was determined by gas chromatography taking CH_4_ as an internal standard. Control experiments suggested that PTZ alone could not produce any H_2_ at all (**Figure** **1**b) and the as‐synthesized CdSe QDs evolved trace amount of H_2_ either under visible‐light irradiation (Figure 1b). However, introduction of PTZ onto the surface of CdSe QDs significantly enhanced the rate of photocatalytic H_2_ evolution to a climax of ≈14 μmol h^−1^, about 40‐fold to that obtained in the absence of PTZ (Figure 1a). Further increasing the amount of PTZ, the rate of H_2_ evolution showed negligible variation and reached a plateau stage, suggesting that the number of PTZ bound to per QD got saturated. The average number of PTZ on per QD was determined to be ≈7.0 according to the characteristic absorption decrease of PTZ in ethanol (see details in Supporting Information). Under the optimal condition, PTZ‐modified CdSe QDs achieved H_2_ photoproduction with an average rate of ≈7500 μmol h^−1^ g^−1^ (Figure 1b), which was comparable to or even better than that of CdSe QD‐hybrids incorporated with an external co‐catalyst, such as NiCl_2_ (Figure S4, Supporting Information). The PTZ molecules were found to keep the stability toward visible‐light irradiation (Figure S3, Supporting Information). During a period of 10 h irradiation, a total of ≈147 μmol H_2_ was produced, much larger than the amount of surface bound PTZ (≈1.12 μmol), which excluded the possibility of PTZ as a sacrificial reagent.

**Figure 1:**
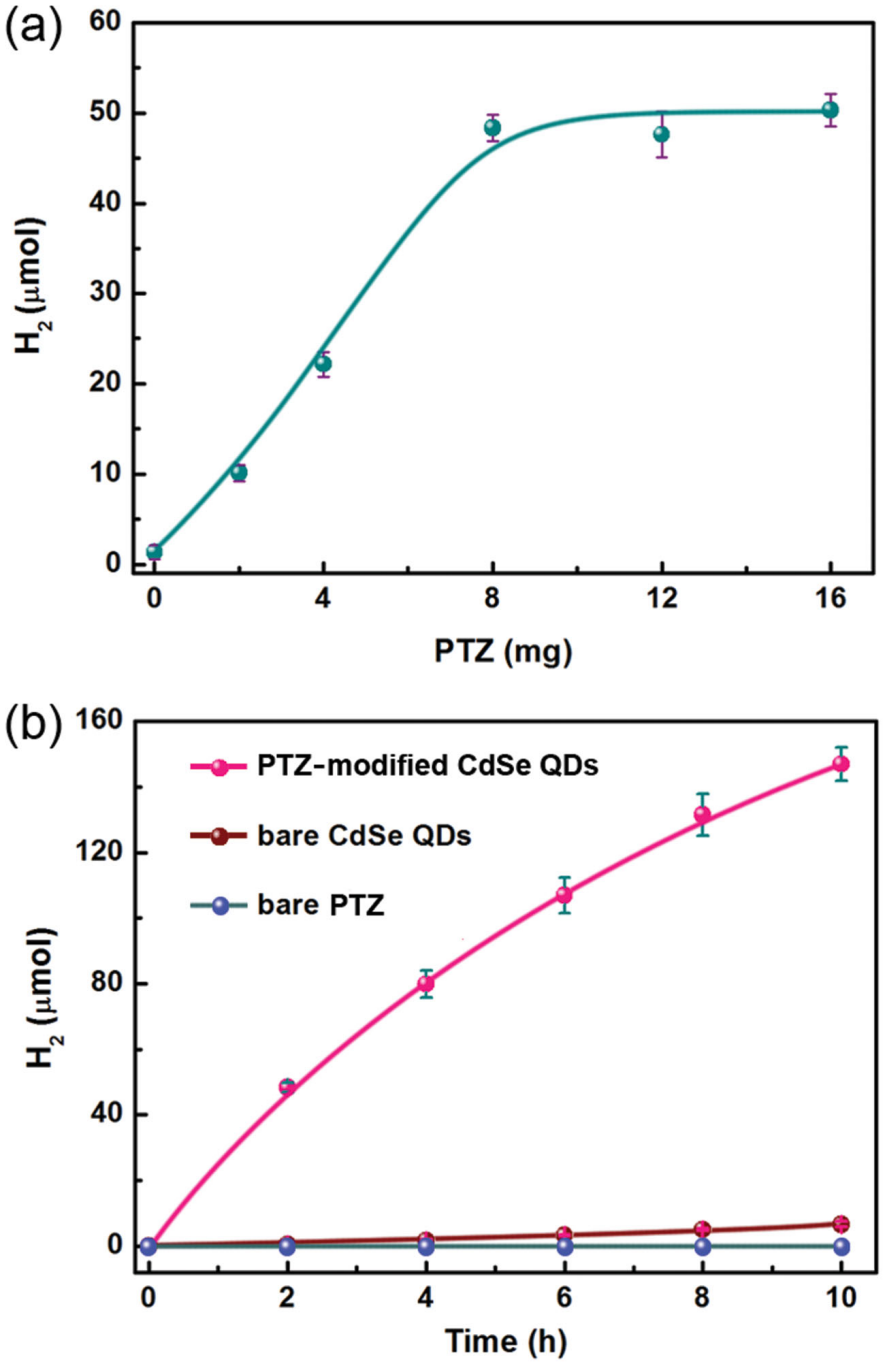
Photocatalytic H_2_ evolution of CdSe QDs: a) PTZ loading amount related H_2_ evolution in 2.0 h; b) H_2_ evolution of bare CdSe QDs, PTZ‐modified CdSe QDs (1.6 × 10^−5^ mol L^−1^) and bare PTZ (1.1 × 10^−4^ mol L^−1^) from 10.0 mL ascorbic acid aqueous solution (0.2 m) at pH 4.0 irradiated by a 500 W high pressure mercury‐lamp (≈100 mW cm^−2^) with a cut‐off filter to remove UV‐light at room temperature. Error bars represent mean ± s.d. of three independent experiments.

Because light absorption of QDs, ability of proton reduction, pH value of solution and type of sacrificial donor remained unchanged before and after introducing PTZ, the significant enhancement of H_2_ photoproduction could be attributed to better charge separation of CdSe QDs by introducing PTZ to achieve efficient hole extraction. PTZ‐mediated hole transfer could be directly confirmed by spectroscopic and spectroelectrochemical studies. Upon introduction of PTZ onto QD surface, the luminescence of CdSe QDs was almost completely quenched (Figure S1e, Supporting Information). Luminescent quenching of CdSe QDs could be either due to energy transfer or charge transfer from photoexcited CdSe QDs to surface bound PTZ. Energy transfer is not possible because there is no overlap between PTZ absorption and QD emission, as presented in Figure S1b,d (Supporting Information). According to the energy alignment of valence band of CdSe QDs and HOMO level of PTZ (Figure S5, Supporting Information), it could be concluded that only hole transfer from CdSe QDs to PTZ was thermodynamically feasible in our system. The luminescence quenching of CdSe QDs could be therefore attributed to the hole extraction of QDs by PTZ. Importantly, the hole transfer from excited CdSe QDs to surface bound PTZ was found to occur in hundreds of picoseconds (≈300 ps),[[qv: 12a]],^14^ much faster than that of hole transfer directly from CdSe QDs to ascorbic acid (in tens of nanoseconds, with a rate constant of 1.5 × 10^9^ s^−1^ m ^−1^) (Figure S6, Supporting Information). The above mentioned kinetics supported a more efficient hole‐extraction pathway from excited CdSe QDs to surface‐bound PTZ ligands rather than ascorbic acid in solution. To prove that the extracted holes in PTZ could be further captured by ascorbic acid, we prepared the oxidized PTZ species with external bias of +0.9 V versus NHE by spectroelectrochemical technique (Figure S7, Supporting Information). With the addition of ascorbic acid, the absorption of oxidized PTZ species disappeared immediately, suggesting that the successive hole transfer from CdSe QDs to PTZ, and then to ascorbic acid could proceed smoothly.

The large difference of solar H_2_ evolution in the presence and absence of PTZ demonstrates the vital importance of surface ligands in influencing the interfacial hole‐transfer of CdSe QDs. As depicted in **Scheme** **1**, the hole transfer from QDs to hole‐accepting ligands, PTZ (*k*_HT2_), is much faster than that directly to sacrificial reagent in solution, ascorbic acid (*k*_HT1_), which in turn slows down the charge recombination process (*k*_CR_). As the introduction of PTZ presents little influence on the light absorption (*I*_ABS_) of CdSe QDs and the electron transfer (*k*_ET_) to surface cadmiums, we believe that the rapid removal of photogenerated holes from CdSe QDs can provide an efficient pathway to release electrons from the Coulomb drag of holes and yield long‐lived charge separation. As the exciton binding energy and electron‐hole recombination become more apparent in nanoparticles,^15^ introduction of saturated amount of PTZs on CdSe QDs could also lead to multiple exciton dissociation via ultrafast hole extraction from QDs to surface bound hole acceptors, both of which resulted in improving the solar‐to‐fuel conversion efficiency of CdSe QDs for solar H_2_ evolution.

**Scheme 1:**
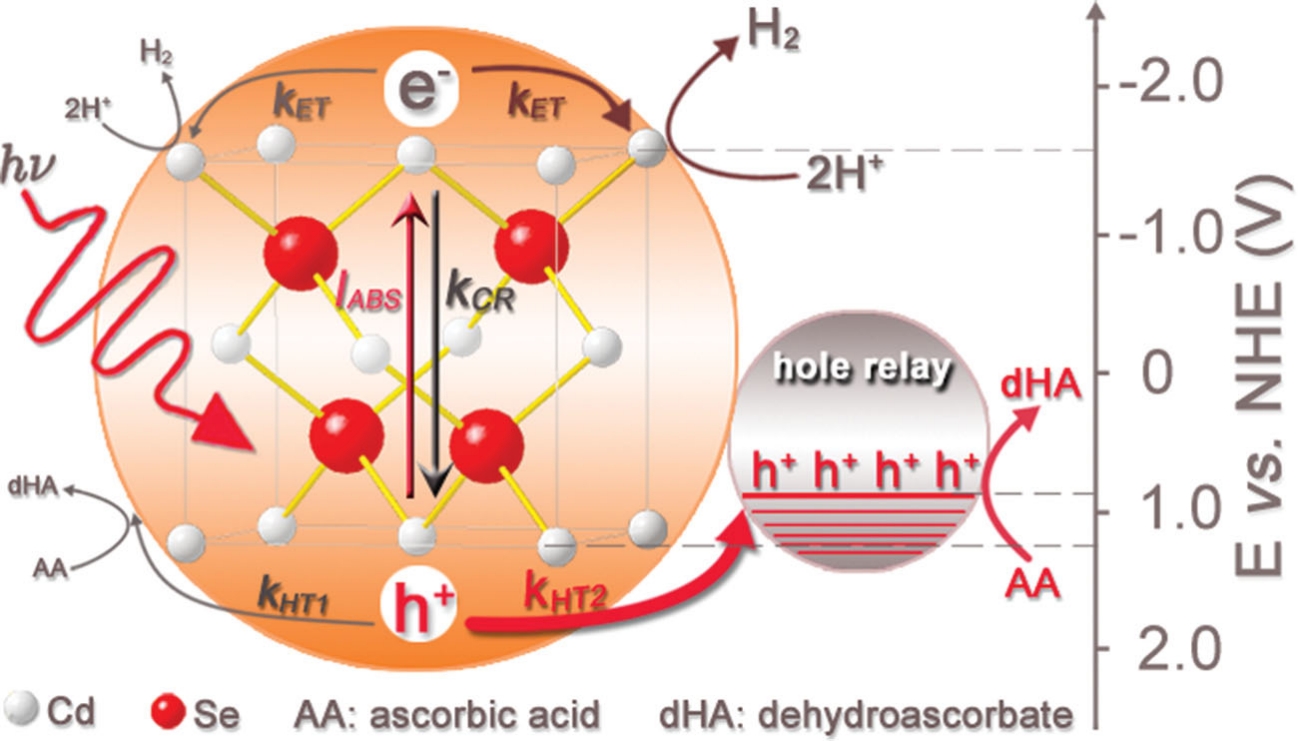
Schematic illustration of the solar H_2_ evolution of CdSe QDs in the presence and absence of hole‐accepting ligands (HAL) and the corresponding interfacial charge transfer processes.

More importantly, we found hole‐accepting ligand modification could dramatically improve the PEC H_2_ evolution of CdSe QD‐sensitized photocathode either. Sample electrodes were fabricated by chemical adsorption of CdSe QDs on NiO/FTO substrates (see details in Supporting Information), in which NiO with a valence band of +0.54 V versus NHE could work as a hole acceptor.^16^ PTZ‐modified CdSe QDs electrode gave identical absorption with bare CdSe QDs counterpart in the UV–vis diffuse reflectance spectra, indicating that similar amount of CdSe QDs had been successfully tethered onto the electrode (Figure S8, Supporting Information). High‐resolution TEM image further confirmed the anchoring of CdSe QDs on NiO (Figure S9a, Supporting Information). Presence of PTZ on CdSe QDs was directly inferred by elemental mapping of uniformly dispersed nitrogen from the energy dispersive X‐ray spectroscopy, because nitrogen only existed in PTZ molecules (Figure S9b, Supporting Information). The identical Raman signals from PTZ‐modified CdSe QDs electrode and pure PTZ, respectively, further confirmed the successful introduction of PTZ on CdSe QDs (Figure S10, Supporting Information).

PEC measurements were performed in a three‐electrode system, in which the sample electrode was used as a photocathode, Ag/AgCl (3.0 m KCl) was as the reference electrode, platinum was as the counter electrode, and 0.1 m Na_2_SO_4_ aqueous solution was as the electrolyte. Similar to solar H_2_ evolution in aqueous solution, the presence of PTZ molecules on the surface of CdSe QDs had a significant influence on the PEC performance. Apparently, introduction of PTZ onto CdSe QDs could enhance the transient photocurrent responses of CdSe‐sensitized photocathode (Figure S11, Supporting Information). Further increasing the amount of PTZ led to negligible variation of current density, probably due to the saturated PTZ loading on per individual QD. *J*–*V* curves of PTZ‐modified CdSe QDs electrode collected under chopped illumination by a 300 W Xe‐lamp with a UV cut‐off filter (*λ* > 400 nm) revealed photo­currents reaching about −180 μA cm^−2^ at an applied potential of −0.1 V versus NHE, about 2.5‐fold to that of bare CdSe QDs counterpart (−70 μA cm^−2^) (**Figure** **2**a). Under the same condition, NiO/FTO electrode gave negligible photocurrents, indicating the responsive CdSe QDs toward light (Figure 2a). Linear sweep voltammetry measurement revealed that PTZ‐CdSe QD/electrode brought about photocurrents under visible‐light illumination with an onset potential of ≈0.37 V versus NHE, larger than that obtained in the absence of PTZ (Figure S12, Supporting Information). IPCE plots of both electrodes matched well with the UV–vis absorption spectrum of CdSe QDs, and reached a maximum of ≈10.3% for PTZ‐CdSe QD‐sensitized photocathode in the realm of visible‐light irradiation, about 2.5‐fold to that obtained in the absence of PTZ (≈4.3%) (Figure 2b). The dramatically improved photocurrent response and IPCE value directly confirmed that PTZ could improve the charge‐separation efficiency through hole‐extraction of CdSe QDs. Similarly, PTZ could increase the photocurrent response of CdS QD‐electrode a lot, but kept that of CdTe counterpart unchanged (Figure S13, Supporting Information). These observations were related to the fact that valence band potentials of CdS QDs and CdTe QDs are ≈1.6 V and ≈0.3 V versus NHE, respectively.^17^ The process of hole transfer from CdS QDs to PTZ is thermodynamically feasible, but hole extraction of CdTe QDs by PTZ is hard to occur (Figure S14, Supporting Information). These results further confirmed that the improved PEC performance of CdSe QDs‐photocathode was derived from the hole‐extraction of surface bound PTZ.

**Figure 2:**
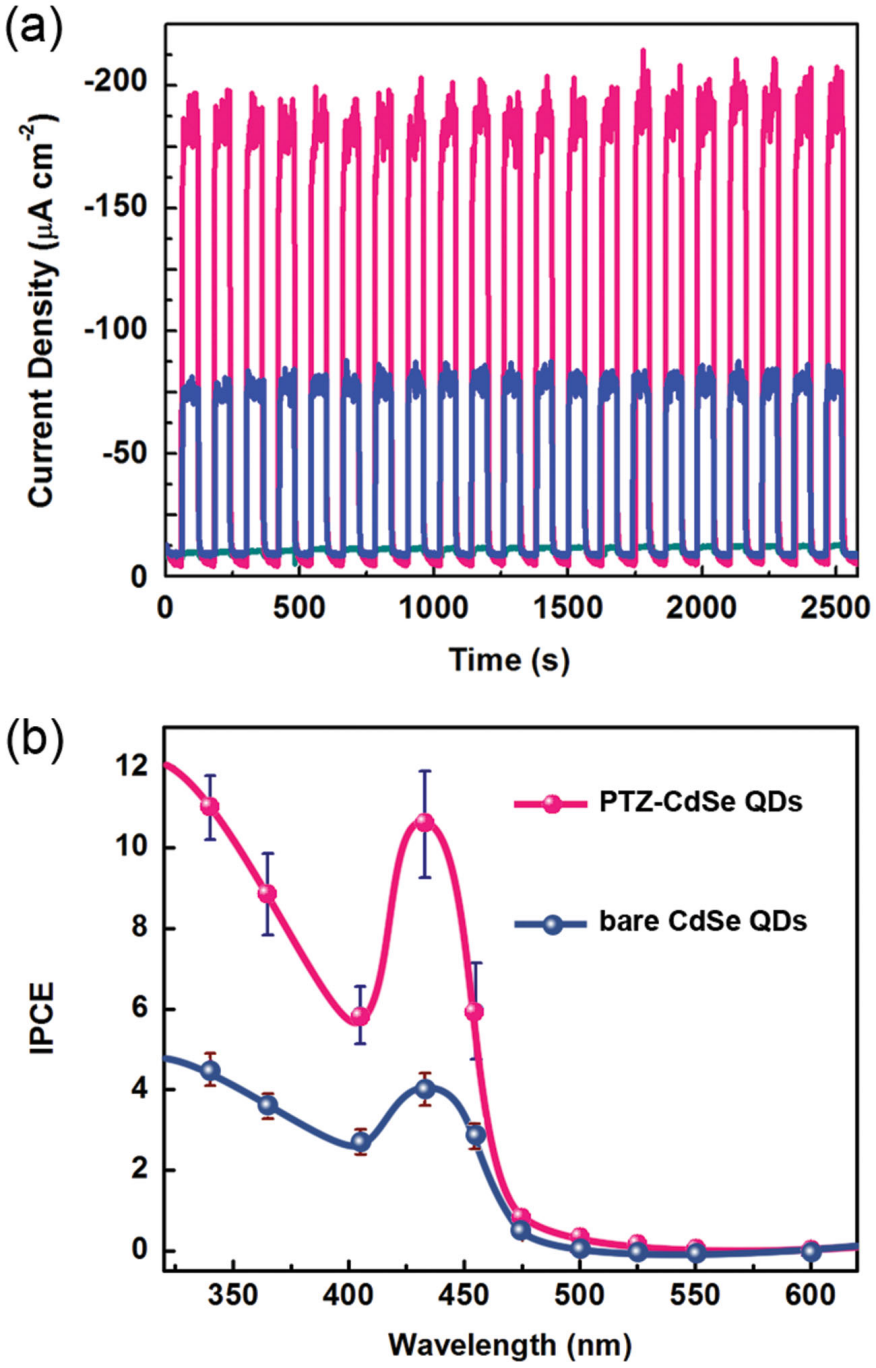
PEC measurements: a) transient photocurrent responses to chopped visible‐light irradiation of NiO electrode (cyan line), bare CdSe QDs electrode (blue line) and PTZ‐modified CdSe QDs electrode (pink line); b) IPCE spectra of bare CdSe QDs electrode (blue line) and PTZ‐modified CdSe QDs electrode (pink line). Error bars represent mean ± s.d. of three independent experiments.

Impressively, PEC H_2_ evolution performance from PTZ‐modified CdSe QD‐sensitized electrode increased dramatically in the absence of any sacrificial reagent under visible‐light irradiation and applied bias of −0.1 V versus NHE. Rate of H_2_ production from PTZ‐modified CdSe QDs‐sensitized photocathode was determined to be ≈3000 μmol h^−1^ g^−1^ cm^−2^ based on the amount of CdSe QDs, ≈0.3 mg cm^−2^ obtained from inductively coupled plasma‐atomic emission spectroscopy, bound to the mesoporous NiO film, and thereby leading to a turnover frequency (TOF) of ≈0.2 per second. The activity was also ≈2.5 times that of CdSe QDs electrode in the absence of PTZ (≈1200 μmol h^−1^ g^−1^ cm^−2^) under the same condition (**Figure** **3**a). After visible‐light irradiation for 12.0 h, a total of ≈4.53 μmol molecular H_2_ evolved and 0.90 C charges passed through the external circuit, which gave rise to a Faradic efficiency close to 100%, meaning that almost all photogenerated electrons were consumed for proton reduction. In order to reveal the oxidizing product by photogenerated holes on the counter electrode, electron paramagnetic resonance (EPR) experiment was performed by taking 5,5‐dimethyl‐1‐pyrroline‐N‐oxide (DMPO) as a trapping reagent. Under PEC condition for 1.5 h, a set of signal for typical spin adduct of ^•^OH radicals with DMPO was detected from the three‐electrode system by employing PTZ‐modified CdSe QD‐sensitized photocathode as working electrode under the inert atmosphere (Figure 3c).^18^ The high intensity of EPR signal verified that large amount of ^•^OH radicals had been obtained through water oxidation by photogenerated holes. This amazing result demonstrated that our designated PEC system could efficiently achieve water splitting in the absence of any sacrificial electron donors, co‐catalysts, protecting layers and buffer solutions from neutral water. As water oxidation has been regarded as the bottleneck of water splitting, these observations presented would provide guidance for fabrication of highly efficient, clean and earth‐abundant PEC devices to split water by using visible light.^19^ Control experiments demonstrated that both visible‐light irradiation and applied bias were necessary for realizing PEC water oxidation (Figure S15, Supporting Information). Long‐time measurement presented negligible decrement of activity as long as 20 h (Figure 3b). This strongly supported the avoidance of accumulating holes that would have caused the photocorrosion of CdSe QDs.[[qv: 17a]] As the potentials needed for water oxidation and proton reduction were strongly pH dependent,^20^ we found that the transient photocurrent responses of PTZ‐modified CdSe QDs‐electrode were also pH‐dependent (Figure S16, Supporting Information).

**Figure 3:**
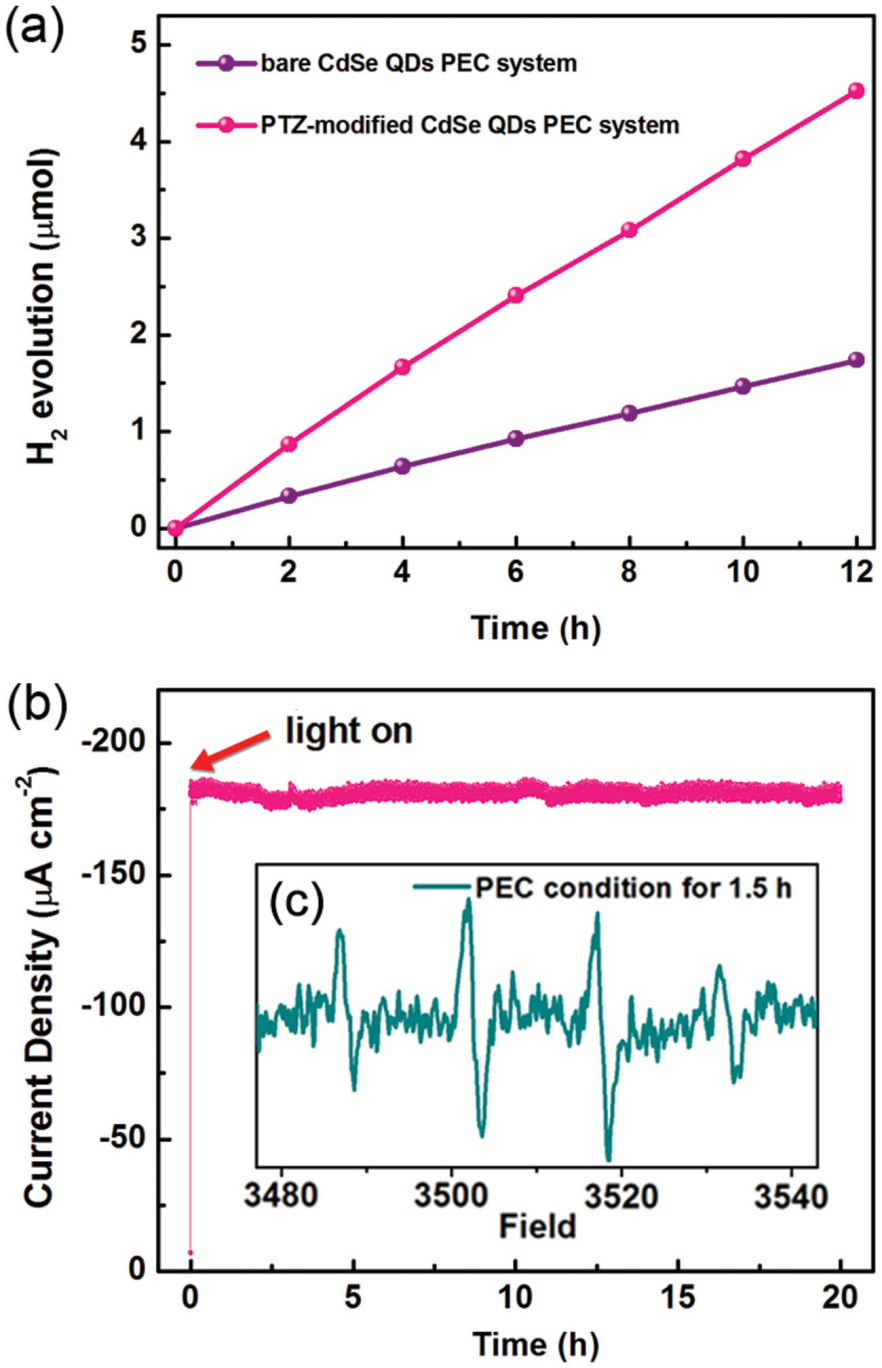
a) Time courses of H_2_ evolution in the three‐electrode system by taking bare CdSe QDs electrode and PTZ‐modified CdSe QDs electrode as the working electrode, respectively; b) long time *J*–*t* curve in 20.0 h test; c) EPR signal obtained from the three‐electrode PEC system by using 0.02 m DMPO as a trapping agent under visible‐light irradiation (*λ* > 400 nm) and −0.1 V versus NHE for 1.5 h.

PTZ modification could achieve ultra‐fast and efficient hole extraction of CdSe QDs, leading to significant enhancement of the PEC H_2_ evolution of CdSe QD/photocathode. In order to evaluate the influence of hole‐accepting ligands on the hole‐extraction process of QDs, we carried out the transient photocurrent measurements by using PTZ derivatives with different substituents. It could be observed that CdSe QDs‐sensitized photocathodes in the presence of PTZ derivatives with electron‐donating substituents gave much larger cathodic photocurrents than those with electron‐withdrawing groups (**Figure** **4**). The higher increase of photocurrent could be assigned to either higher adsorption of hole‐accepting ligands on CdSe QDs or better hole‐extraction efficiency. Although the different solubility of PTZ derivatives led to different saturated concentrations in ethanol, the surface coverage of hole‐accepting molecules should only be determined by the number of available adsorption sites on CdSe QDs. Because the number of adsorption sites did not change by using a single synthetic batch of CdSe QDs for all of the experiments, it is reasonable to attribute the higher photocurrent of 10‐methylphenothiazine to better hole extraction, which might be due to either larger free energy (−Δ*G*) for hole transfer from QDs to adsorbates or better electron‐donating ability of methyl group. Also, similar photocurrent variation had been observed by introducing thiophene and its derivatives onto the surface of CdSe QDs (Figure S17, Supporting Information).

**Figure 4:**
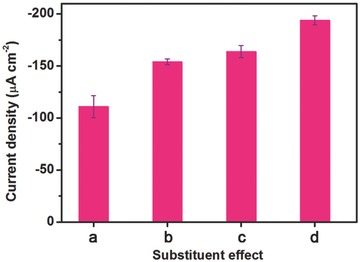
Transient photocurrent responses of CdSe QD‐sensitized photocathodes modified by PTZ derivatives: a) phenothiazine‐10‐carbonyl chloride, b) 10‐propionylphenothiazine, c) phenothiazine, and d) 10‐methylphenothiazine. Error bars represent mean ± s.d. of three independent experiments.

In conclusion, we have established a facile approach to improve the efficiency of solar‐to‐fuel conversion from QD‐based artificial photosystems, which is integration of hole‐accepting ligands onto the surface of CdSe QDs. Simply introduction of PTZ onto the surface of CdSe QDs can dramatically increase the rate of H_2_ evolution from CdSe QDs in aqueous solution and under PEC condition. The facilitated interfacial hole extraction process achieved by PTZ has been verified by transient photocurrent responses, IPCE measurement, spectroscopic and spectroelectrochemical studies. Obviously, this strategy provides a guidance to overcome the hole‐transfer limitation and offers a new opportunity to make artificial photo­synthetic H_2_ evolution practically viable. Ongoing efforts are focused on providing quantitative analysis of the hole transfer at nanoscale interfaces, ameliorating the interaction between ligands and QDs, and finally targeting to achieve total water splitting with higher efficiency.

## Supporting information

As a service to our authors and readers, this journal provides supporting information supplied by the authors. Such materials are peer reviewed and may be re‐organized for online delivery, but are not copy‐edited or typeset. Technical support issues arising from supporting information (other than missing files) should be addressed to the authors.

Supplementary
