# Supplementary material for: Hole‐Accepting‐Ligand‐Modified CdSe QDs for Dramatic Enhancement of Photocatalytic and Photoelectrochemical Hydrogen Evolution by Solar Energy
Source: Adv Sci (Weinh). 2015 Dec 2;3(4):1500282. doi: 10.1002/advs.201500282 (PMC5063123; doi:10.1002/advs.201500282)
Supplement: Supplementary file 1 — Supplementary [file ADVS-3-0o-s001.pdf]

Copyright WILEY-VCH Verlag GmbH & Co. KGaA, 69469 Weinheim, Germany, 2015.

ADVANCED SCIENCE
Open Access

Supporting Information

for *Adv. Sci.*, DOI: 10.1002/advs.201500282

Hole-Accepting-Ligand-Modified CdSe QDs for Dramatic Enhancement of Photocatalytic and Photoelectrochemical Hydrogen Evolution by Solar Energy

*Xu-Bing Li, Bin Liu, Min Wen, Yu-Ji Gao, Hao-Lin Wu, Mao-Yong Huang, Zhi-Jun Li, Bin Chen, Chen-Ho Tung, and Li-Zhu Wu**

Supporting Information

Hole-Accepting-Ligand Modified CdSe QDs for Dramatic Enhancement of Photocatalytic and Photoelectrochemical Hydrogen Evolution by Solar Energy

*Xu-Bing Li, Bin Liu, Min Wen, Yu-Ji Gao, Hao-Lin Wu, Mao-Yong Huang, Zhi-Jun Li, Bin Chen, Chen-Ho Tung, and Li-Zhu Wu**

Key Laboratory of Photochemical Conversion and Optoelectronic Materials,

Technical Institute of Physics and Chemistry, Chinese Academy of Sciences, Beijing

100190, P. R. China.

**Corresponding Author. E-mail:* lzwu@mail.ipc.ac.cn.

Dr. Xu-Bing Li, Dr. Bin Liu contributed equally to this work.

A Table of Contents

1. Chemicals
2. Experimental section
3. Characterization
4. Steady-state spectra
5. High resolution TEM image
6. FTIR spectra
7. Solar H_2 evolution
8. Energy level alignment
9. Emission quenching of CdSe QDs by ascorbic acid
10. Spectroelectrochemical experiment
11. DRS spectra
12. Characterization of QD-electrodes
13. Raman resonance spectra
14. Influence of PTZ on photocurrent
15. LSV curve
16. Transient photocurrent responses
17. Energy alignment
18. EPR experiment
19. pH effect
20. Transient photocurrent responses
21. References

1. Chemicals

Phenothiazine (99%), Thioglycollic acid (Alfa aesar, 99%), selenium powder (Aldrich, about 200 mesh), Ascorbic acid (Alfa aesar, 99%), glutathione (Acros, 98%), carbazole (99%), 2-Methylthiophene (99%), $CdCl_2 \cdot 5/2H_2O$ (99%), $Zn(OAc)_2$ (99%), $Zn(NO_3)_2$ (99%), Na_2SO_3 (99%), $NiCl_2 \cdot 6H_2O$ and $Na_2S \cdot 9H_2O$ were purchased from Sigma-Aldrich. Other chemicals are of analytical grade and used without further purification unless otherwise noted. Ultrapure water with 18.2 MΩ cm (Mettler Toledo, FE20, China) was used thorough.

2. Experimental section

Synthesis of water-soluble CdSe QDs:[1] briefly, selenium powder（40 mg）was transferred to Na_2SO_3 aqueous solution (189.0 mg in 100 mL). The resulting mixture was then refluxed until the selenium powder dissolved completely to obtain transparent Na_2SeSO_3 solution. Aqueous, colloidal water-soluble CdSe QDs were prepared by mixing a solution of $CdCl_2 \cdot 5/2H_2O$ and stabilizer, thioglycollic acid (TGA), then adjusting the pH to 11.0 with 1.0 M NaOH, and finally adding a measured volume of the above synthesized Na_2SeSO_3 solution; the typical molar ratio of Cd:stablizer:Se was 1.0:1.5:0.25. This mixture was placed in a three-necked flask and deaerated with N_2 bubbling for 30 min.

Preparation of PTZ-modified CdSe QDs: TGA-CdSe QDs aqueous solution (1.3×10^{-4} mol/L; 10.0 mL) were aggregated and precipitated by 0.1 M hydrochloric acid solution. The precipitate was isolated through centrifugation (5000 rpm, 5.0 min) and washed with ultra-pure water for at least three times to remove any residual ligands in solution and stabilizers bound to the surface of QDs. Then, the precipitate was re-dispersed in PTZ ethanol solution (8.0 mg/10 mL). The solution was vigorously stirred and sonicated for 30 min to ensure the maximal binding of PTZs onto the surface of CdSe QDs to obtain PTZ-modified CdSe QDs. After the assembling procedure, the characteristic absorbance of PTZ in ethanol decreased from A_1 (0.8944) to A_2 (0.6681). According to the Lambert-Beer law, the amount of PTZ bound to CdSe QDs was determined to ~1.8104 mg. Herein, the average number of PTZ molecules bound to per QD was determined to ~7.0, which was consistent with the

reported value. PTZ modified CdS QDs and CdTe QDs were prepared by using the same method. The formula of PTZ is $C_{12}H_9NS$, and the molecular structure of PTZ is 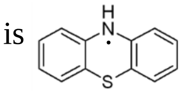

Solar H_2 evolution: typically, the above prepared PTZ-modified CdSe QDs (1.6×10^{-5} mol/L) was re-dispersed in 10.0 mL ascorbic acid aqueous solution (0.2 M) at pH 4.0. The reaction mixture was degassed through bubbling nitrogen for 30 min to remove the residual oxygen gas, and then irradiated with a 500 W high-pressure mercury lamp (ZhongRuiDa Electric Light Factory, Beijing, China) with a cutoff filter to remove light below 400 nm (~100 mW cm^{-2} at flask surface) at room temperature. The molecular H_2 evolved was determined by GC taking CH4 as internal standard. Solar H_2 evolution of bare CdSe QDs and other QDs/Hole-relay assemblies was performed in a similar procedure.

Preparation of NiO/FTO electrode: NiO mesoporous electrode was fabricated by following the reported method.[2] Briefly, an aqueous suspension of commercially available NiO nanoparticles (mean size below 50 nm) was printed on FTO substrate by using a semiautomatic screen printer. FTO substrate printed with aqueous suspension film of NiO was then sintered at 450 °C for 2.0 hour under air atmosphere, and then the NiO mesoporous film electrode was obtained after being cooled to room temperature.

Fabrication of PTZ-modified CdSe QDs electrode: the above prepared PTZ-modified CdSe QDs was dispersed in thioglycolic acid (TGA) (0.23 mmol) aqueous solution (5.0 mL) at pH 11.0 to form a bright-yellow solution (2.0×10^{-4} mol/L). Subsequently, a mesoporous NiO/FTO electrode, prepared according to the reported method, was soaked in the TGA solution for 4.0 hours to ensure the saturated binding of PTZ-modified CdSe QDs on NiO. Then, the PTZ-modified CdSe QDs electrode was washed with ultrapure water for three times and dried in the air. Similar procedures were applied for the preparation of bare CdSe QDs electrode.

IPCE measurement: the experiment was performed in a three-electrode setup by taking the QDs based electrodes as the working electrode, platinum disk as counter electrode, Ag/AgCl (3.0 M KCl) as reference electrode, and 0.1 M aqueous solution of Na_2SO_4 as electrolyte under inert atmosphere. Monochromatic LEDs were

employed as the light source and the intensity of the incident light was measured with a Newport photometer. Hence, the IPCE value could be calculated according to the photocurrent density (J), the wavelength of incident light (λ), and the intensity of the incident light (I).

Determination of the Faradic efficiency of PEC H_2 evolution: the Faradic efficiency was determined in a three-electrode system under a bias of -0.1 V vs NHE and visible light irradiation. In a 12.0 h PEC test, total of ~0.335 C charge carriers passed through the external circuit and simultaneously ~1.74 μmol of molecular H_2 was detected by GC for bare CdSe electrode system. Herein, the Faradic efficiency of H_2 evolution from water splitting was determined to be 100%. Faradic efficiency of PEC H_2 evolution for PTZ-modified CdSe QDs electrode was determined by using the same method, which was also determined to be close to 100%.

3. Characterization

UV-Vis absorption spectra were recorded with a Shimadzu 1601PC spectrophotometer. Photoluminescence measurements were performed at room temperature using a Hitachi 4500 fluorescence spectrophotometer. All optical measurements were performed at room temperature. Diffuse reflectance spectra was carried on a Cary 5000 UV-visible-NIR spectrophotometer. X-ray diffraction pattern was obtained by using Bruker D8 Focus under Cu-K_α radiation. High-resolution transmission electron microscopy was performed on a JEM 2100F (Japan) electron microscope operated at an accelerating voltage of 200 kV. All pH measurements were made with a Model pHS-3C meter (Mettler Toledo FE20, China). Raman resonance spectra were carried out by using Via-Reflex Raman system with a 532 nm excitation wavelength. Scanning electron microscopy and energy dispersive X-ray spectroscopy were obtained by using HITACHI S-4800. Gas chromatography was performed on a TIAN MEI 7890 II GC using a 5 Å molecular sieve column and a thermal conductivity detector. Luminescence decay measurements were performed with a FLSP920 (Edinburgh Instruments Ltd., UK) apparatus with 405 nm laser excitation (pulse width ca.100 ps). Element content was determined by an inductively coupled plasma mass spectroscopy (ICP, Varian 710-ES). Fourier transform infra-red (FTIR) spectroscopy (Excalibur 3100). Photoelectrochemical measurement was carried out in a three-electrode setup with the working electrode of the sample film electrode, counter electrode of platinum disk, and reference electrode of Ag/AgCl (3.0 M KCl). The electrolyte (0.1 M Na_2SO_4, pH = 6.8) was degassed for 120 minutes by flushing high purity argon prior to and during each measurement. For photocurrent measurement, the light source was a 300 W Xe-lamp with a UV cut-off filter unless otherwise stated; the light intensity at the surface of the electrode is ~100 mW cm^{-2}. Prior to measurement, the area of the electrode was fixed by insulating cement and the exposed area was 0.64 cm^2. A Zennium electrochemical workstation (Germany, Zahner Company) was used. The transient photocurrent response to on-off illumination was performed when the sample electrode was applied a constant potential (-0.1 V *vs* NHE). IPCE was measured under monochromatic light irradiation provided by the xenon lamp equipped with band pass filters. The light intensity was tested with a Newport photometer. Electron paramagnetic resonance was performed by using ESP-300 and DMPO as trapping agent under room temperature.

4. Steady-state spectra

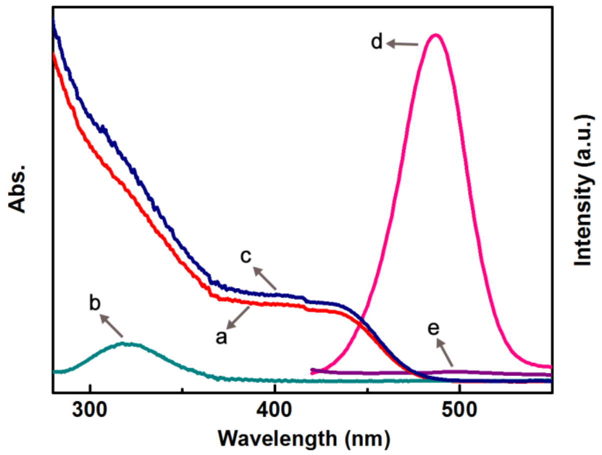

Figure S1. Steady-state absorption spectra of CdSe QDs in water (a), PTZ in ethanol (b) and CdSe QDs in the presence of PTZ in water (c). Steady-state emission spectra of CdSe QD in the absence (d) and presence (e) of PTZ in water (excitation of 400 nm). Because the first excitonic peak for CdSe QDs is around 438 nm and PTZ only absorbs UV light, CdSe QDs can be selectively excited under visible-light irradiation. The absorbtion spectrum of PTZ/CdSe QD complex doesn't show any additional peak, which indicates that formation of charge-transfer complex has been avoided.

5. High-resolution TEM image of CdSe QDs

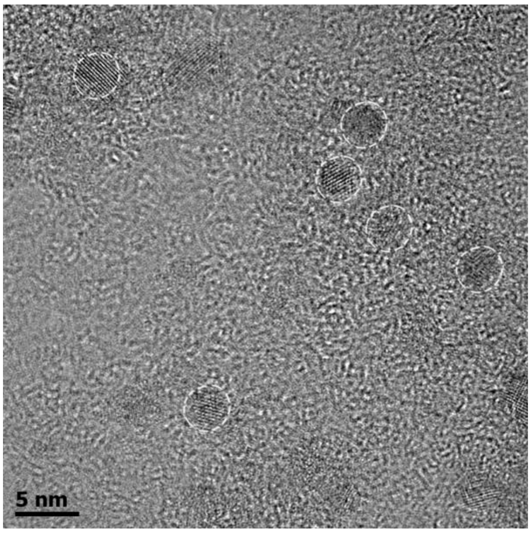

Figure S2. Typical high-resolution TEM image of the as-synthesized CdSe QDs: CdSe nanoparticle is outlined by white cycle, from which we can find that the average size of the as-synthesized CdSe QDs is about 2.0 nm.

6. FTIR spectra

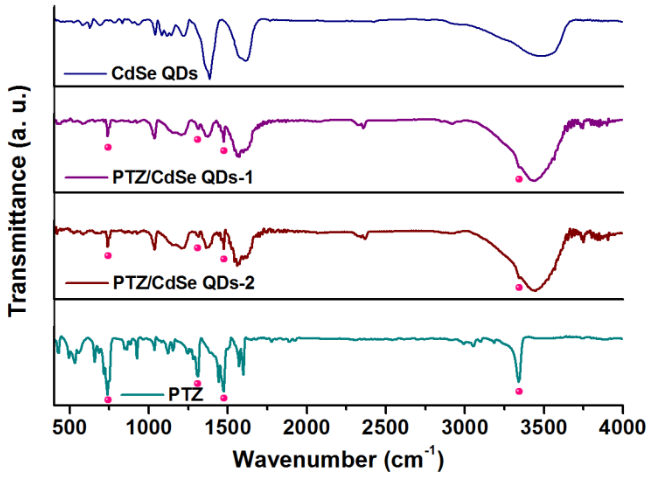

Figure S3. FTIR spectra of bare CdSe QDs, pure PTZ, PTZ modified CdSe QDs before (PTZ/CdSe QDs-1) and after (PTZ/CdSe QDs-2) visible-light irradiation. According to the above results, we could find that the signals of PTZ/CdSe QDs before and after visible-light irradiation were a combination of CdSe QDs and PTZ, which not only verified the successful introduction of PTZ on the surface of CdSe QDs, but also confirmed that PTZ molecules were stable during visible-light irradiation.

7. Photocatalytic H_2 evolution

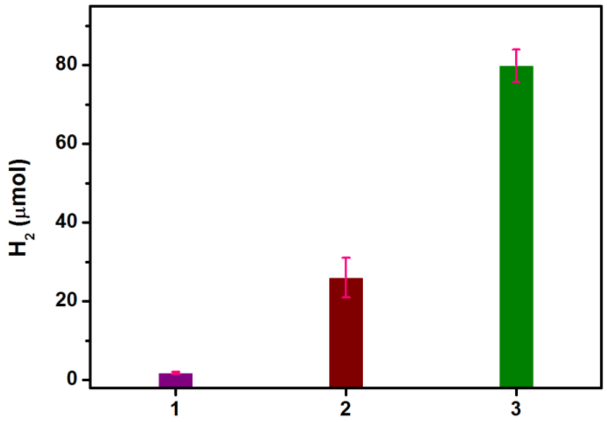

Figure S4. Photocatalytic H_2 evolution from 10 mL ascorbic acid aqueous solution (0.2 M) at pH = 4.0 irradiated by a 500 W high-pressure mercury lamp with a 400 nm cut-off filter under room temperature for 4.0 h. Conditions: (1) bare CdSe QDs (1.6 × 10^{-5} mol/L); (2) CdSe QDs (1.6 × 10^{-5} mol/L) in the presence of 1.0 mg $NiCl_2{\cdot}6H_2O$; and (3) PTZ-modified CdSe QDs (1.6 × 10^{-5} mol/L). Error bars represent mean ±s.d. of three independent experiments.

8. Energy level alignment

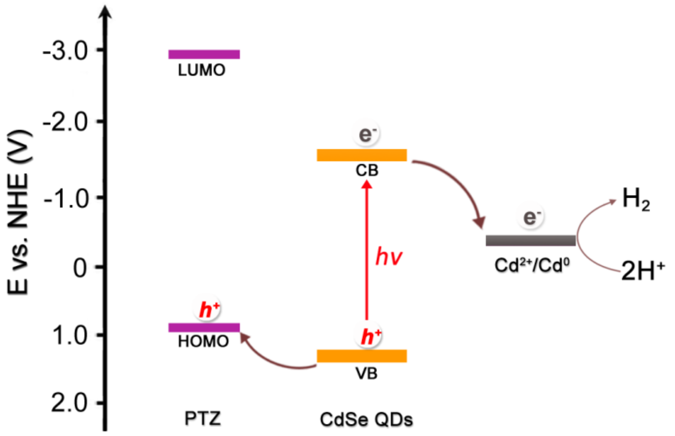

Figure S5. Energy level alignment of CdSe QDs, PTZ molecules and proton reduction catalyst (Cd^{2+}/Cd^{0}), and the processes of interfacial charge transfer.

9. Emission quenching of CdSe QDs by ascorbic acid

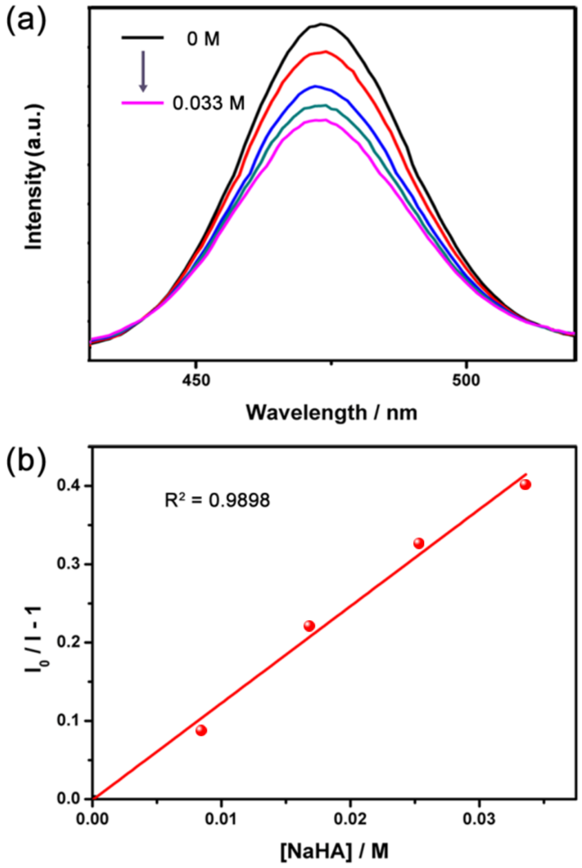

Figure S6. (a) The emission quenching of CdSe QDs (1.6 × 10^{-5} mol·L^{-1}) with adding of different amounts of NaHA at pH 4.0; and (b) the corresponding Stern-Volmer equation of the emission quenching of CdSe QDs during adding of NaHA. (400 nm excitation)

10. Spectroelectrochemical experiment

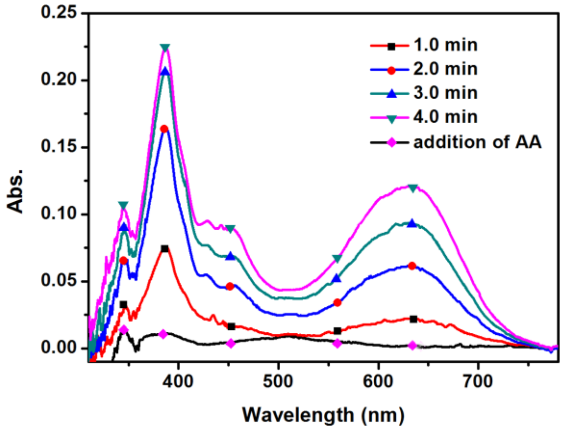

Figure S7. Spectroelectrochemical absorption spectra of oxidized PTZ ($PTZ^{\bullet+}$) in 2.5 mL mixture of CH_3CN/H_2O (v/v = 1/1) by oxidation of PTZ (2.0×10^{-4} mol L^{-1}) at an applied potential of +0.9 V vs NHE.

11. DRS spectra

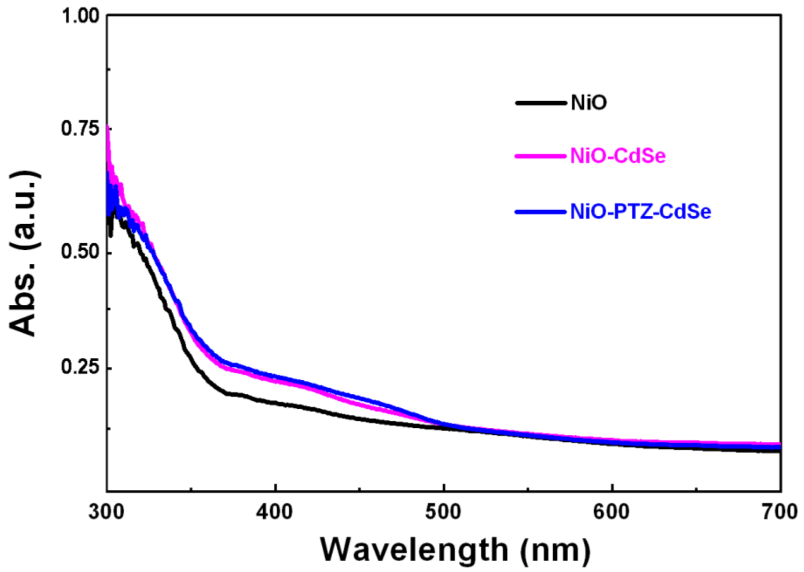

Figure S8. DRS spectra of mesoporous NiO film electrode, NiO-CdSe QDs electrode and NiO-PTZ-modified CdSe QDs electrode. According to the UV-Vis diffuse reflectance spectra of bare NiO, the band gap of NiO is estimated to be ~3.5 eV, which is highly consistent with the literature reported value.[3]

12. Characterization of QD-electrodes

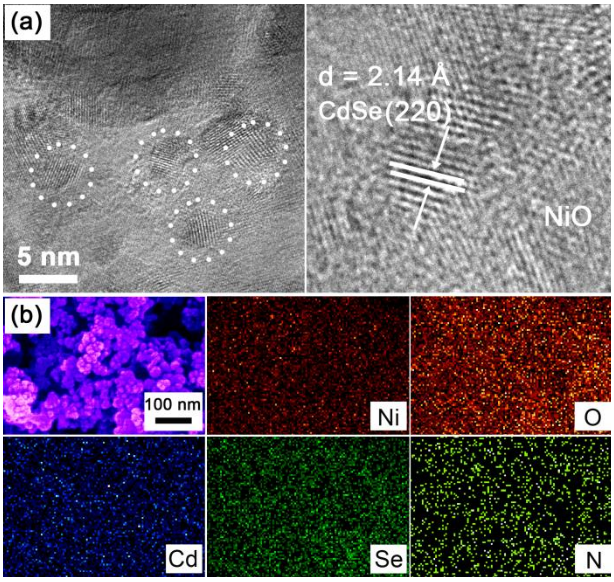

Figure 9. Characterization of PTZ-modified CdSe QDs electrode: (a) TEM and corresponding HRTEM images of the sample electrode, CdSe QDs are depicted with white circles; (b) SEM image and corresponding elemental mapping of nickel, oxygen, cadmium, selenium and nitrogen by EDX spectroscopy, respectively.

13. Raman resonance spectra

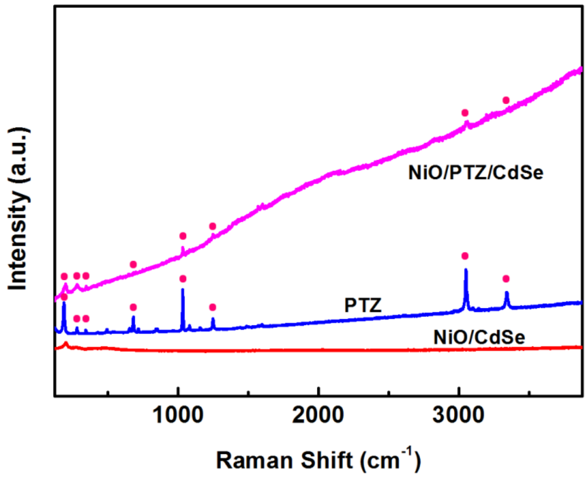

Figure S10. Raman resonance spectra are used to verify the successful introduction and well preservation of the structure of PTZ molecules on the surface of CdSe QDs. The electrode of PTZ-modified CdSe QDs gave the indentical signals as obtained from pure PTZ under the same condition, which indicated the presence of PTZ on the surface of CdSe QDs. (532 nm laser excitation)

14. The influence of PTZ on photocurrent

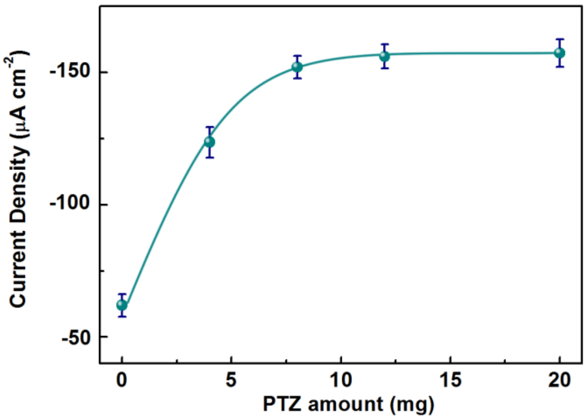

Figure S11. The variation of photocurrent by changing the number of PTZ on the surface of CdSe QDs. The number of PTZ on per QD was controlled by changing the concentration of PTZ in ethanol during the process of preparing PTZ-modified CdSe QDs through ligands exchange approach.

15. LSV curve

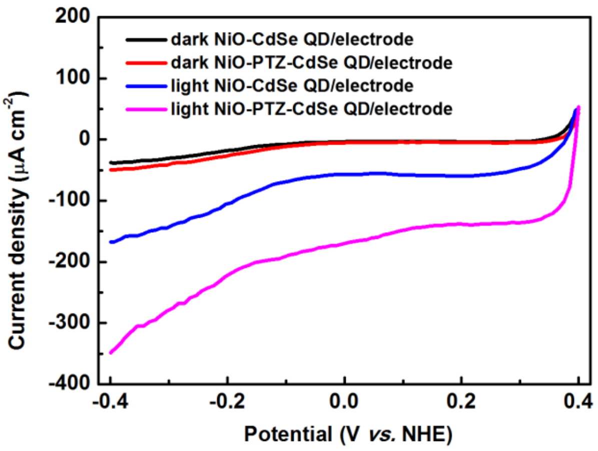

Figure S12. LSV curves (5.0 mV/s) of CdSe QD/electrode and PTZ-modified CdSe QD/electrode obtained in the dark and under visible-light irradiation (~100 mW cm^{-2}) by taking the sample photoelectrode (~0.64 cm^2) as working electrode, Ag/AgCl (3.0 M KCl) as reference electrode, platinum sheet as counter electrode (5 mm × 20 mm), and 0.1 M Na_2SO_4 as electrolyte under 300 W Xe lamp illumination (~100 mW cm^{-2}) with a UV filter to remove the light below 400 nm.

16. Transient photocurrent responses

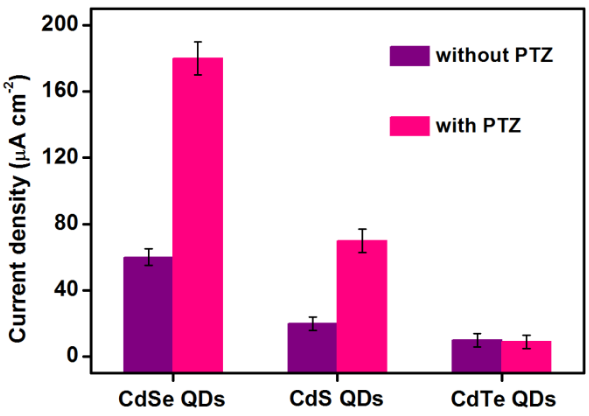

Figure S13. Transient photocurrent responses to visible-light irradiation of CdSe QD-sensitized electrode, CdS QD-sensitized electrode, and CdTe QD-sensitized electrode in the absence and presence of PTZ in neutral aqueous solution, respectively. The valence band of CdS QDs is more positive than the HOMO level of PTZ, hole transfer from QDs to PTZ is thermodanymically feasible. However, the valence band of CdTe QDs is more negative than HOMO level of PTZ, hole extraction is hard to occur.[4]

17. Energy alignment

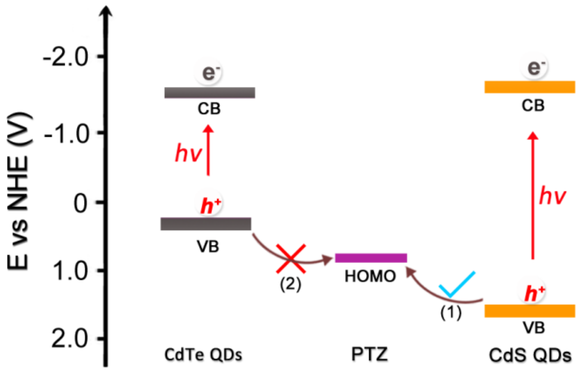

Figure 14. The energy alignment of CdS QDs, CdTe QDs and PTZ molecules. Hole transfer (1) from CdS QDs to PTZ is thermodynamically feasible, but hole transfer (2) from CdTe QDs to PTZ is forbidden.

18. EPR experiment

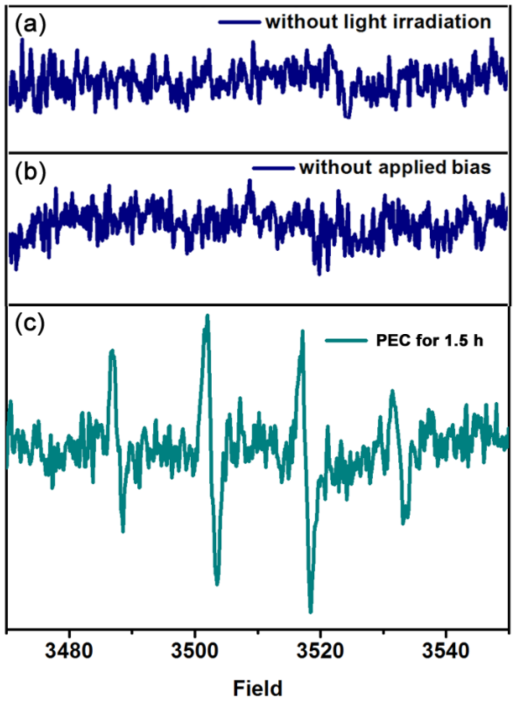

Figure S15. EPR spectra obtained from the three-electrode PEC system of PTZ-modified CdSe QDs electrode by using 0.02 M DMPO as a trapping agent: (a) without visible-light irradiation; (b) without applied bias (direct putting the PTZ-modified CdSe QDs electrode in 0.1 M Na_2SO_4 aqueous solution); and (c) PEC condition ($\lambda > 400$ nm and applied bias -0.1 V vs NHE) for 1.5 h.

19. pH effect

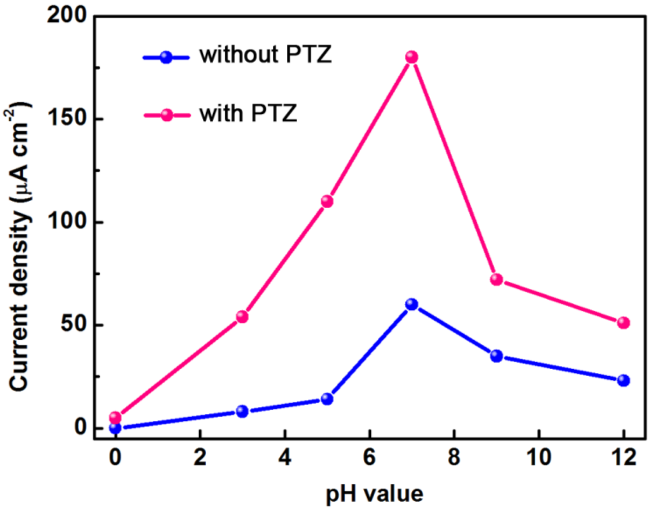

Figure S16. Transient photocurrent responses of CdSe QD-photocathodes in the presence and absence of PTZ at different pH values.

20. Transient photocurrent responses

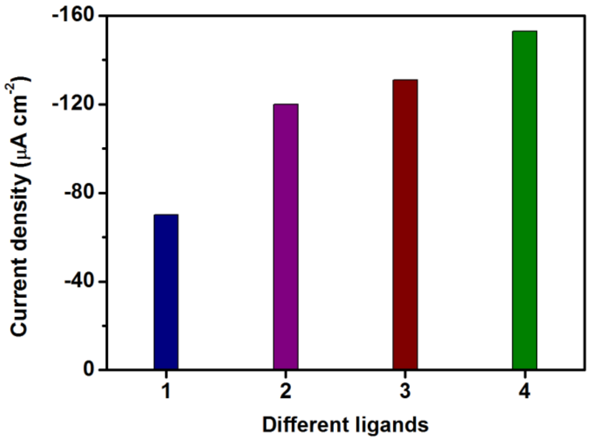

Figure S17. Transient photocurrent responses of different ligands modified CdSe QD-photocathodes: thioglycollic acid (1), 2-Chlorothiophene (2), thiophene (3), and 3-Methoxythiophene (4) under the same conditions.

21. References

[1] a) H.-Y. Han, Z.-H. Sheng, J.-G. Liang, *Mater. Lett.* **2006**, *60*, 3782; b) K. Palaniappan, C. Xue, G. Arumugam, S. A. Hackney, J. Liu, *Chem. Mater.* **2006**, *18*, 1275; c) Z.-J. Li, J.-J. Wang, X.-B. Li, X.-B. Fan, Q.-Y. Meng, K. Feng, B. Chen, C.-H. Tung, L.-Z. Wu, *Adv. Mater.* **2013**, *25*, 6613.

[2] A. Nattestad, A. J. Mozer, M. K. R. Fischer, Y. B. Cheng, A. Mishra, P. Bauerle, U. Bach, *Nat. Mater.* **2010**, *9*, 31.

[3] Z. Zhu, Y. Bai, T. Zhang, Z. Liu, X. Long, Z. Wei, Z. Wang, L. Zhang, J. Wang, F. Yan, S. Yang, *Angew. Chem. Int. Ed.* **2014**, *53*, 12571.

[4] a) F. Wang, W.-G. Wang, X.-J. Wang, H.-Y. Wang, C.-H. Tung, L.-Z. Wu, *Angew. Chem. Int. Ed.* **2011**, *50*, 3193; b) J.-J. Wang, Z.-J. Li, X.-B. Li, X.-B. Fan, Q.-Y. Meng, S. Yu, C.-B. Li, J.-X. Li, C.-H. Tung, L.-Z. Wu, *ChemSusChem* **2014**, *7*, 1468.
